# Effects of High-Intensity Interval vs. Moderate-Intensity Continuous Training on Cardiac Rehabilitation in Patients With Cardiovascular Disease: A Systematic Review and Meta-Analysis

**DOI:** 10.3389/fcvm.2022.845225

**Published:** 2022-02-23

**Authors:** Tian Yue, Yan Wang, Hui Liu, Zhaowei Kong, Fengxue Qi

**Affiliations:** ^1^School of Sports Medicine and Rehabilitation, Beijing Sport University, Beijing, China; ^2^China Institute of Sport and Health Science, Beijing Sport University, Beijing, China; ^3^Faculty of Education, University of Macau, Macau, China; ^4^Sports, Exercise and Brain Sciences Laboratory, Beijing Sport University, Beijing, China

**Keywords:** cardiovascular disease, cardiac rehabilitation, high-intensity interval training, peak oxygen uptake, cardiorespiratory fitness, moderate-intensity continuous training

## Abstract

**Background:**

Studies have shown that high-intensity interval training (HIIT) is superior to moderate-intensity continuous training (MICT) for increasing peak oxygen uptake (VO_2peak_) and reducing cardiovascular disease (CVD) and mortality. To our knowledge, previously published systematic reviews have neither compared different HIIT models with MICT nor investigated intervention frequencies of HIIT vs. MICT for purposes of improving cardiorespiratory fitness in patients with CVD.

**Objective:**

The purpose of this meta-analysis was to compare the effects of different training models, intervention frequencies and weeks of HIIT vs. MICT on changes in cardiorespiratory fitness during cardiac rehabilitation (CR).

**Methods:**

A systematic search was carried out for research articles on randomized controlled trials (RCTs) indexed in the PubMed, Cochrane Library, Web of Science, Embase and Scopus databases for the period up to December 2021. We searched for RCTs that compared the effect of HIIT vs. MICT on cardiorespiratory fitness in patients with CVD.

**Results:**

Twenty-two studies with 949 participants (HIIT: 476, MICT: 473) met the inclusion criteria. Sensitivity analysis revealed that HIIT increased VO_2peak_ more than MICT (MD = 1.35). In the training models and durations, there was a greater increase in VO_2peak_ with medium-interval HIIT (MD = 4.02) and more than 12 weeks duration (MD = 2.35) than with MICT. There were significant improvements in VO_2peak_ with a HIIT frequency of 3 times/week (MD = 1.28). Overall, one minor cardiovascular and four non-cardiovascular adverse events were reported in the HIIT group, while six non-cardiovascular adverse events were reported in the MICT group.

**Conclusion:**

HIIT is safe and appears to be more effective than MICT for improving cardiorespiratory fitness in patients with CVD. Medium-interval HIIT 3 times/week for more than 12 weeks resulted in the largest improvement in cardiorespiratory fitness during CR.

**Systematic Review Registration:**

https://www.crd.york.ac.uk/prospero/display_record.php?ID=CRD42021245810, identifier: CRD42021245810.

## Introduction

Cardiovascular disease (CVD) is responsible for more deaths than any other illness worldwide, and the past decade has witnessed a 12.5% increase in deaths, accounting for 1/3 of the global total (1). The increasing incidence of CVD has increased its financial burden (2). Cardiac rehabilitation (CR) is a promising therapeutic approach to secondary prevention of CVD (3). It includes health education, lifestyle changes, social-psychological support, and supervised exercise (4). Exercise-based CR not only reduces the traditional cardiovascular risk factors (hypertension, hyperlipidemia, diabetes, and obesity) (5), but also cardiovascular risk from conditions such as chronic systemic inflammation (6), which has gradually emerged as a risk factor for CVD (7). Exercise is associated with beneficial anti-inflammatory effects, reduced serum levels of C-reactive protein (CRP) in healthy individuals (8) and improved cardiac output (9), stroke volume (9), and vascular endothelial function (6) as well as reduced heart rate variability (10) in patients with CVD. Exercise-based CR improves cardiorespiratory fitness in patients with CVD (5). Peak oxygen uptake (VO_2peak_), as the gold standard for evaluating cardiorespiratory fitness, has been identified as an important predictor of CVD and all-cause mortality (11). VO_2peak_ is a basic element for controlling CVD all-cause risk factors such as diabetes, dyslipidemia and obesity. Some studies have shown that CVD all-cause mortality decreases by 8–17% when individual cardiorespiratory fitness increases by one metabolic equivalent (12, 13).

Moderate-intensity continuous training (MICT) is regarded as a successful approach to CR because of its efficacy and safety (14–16). Some studies found that MICT can reduce cardiovascular risk and cardiovascular mortality (17, 18). MICT entails longer durations of moderate-intensity continuous aerobic activity, maintaining an intensity between 60 and 80% (VO_2peak_ or reserve heart rate). High-intensity interval training (HIIT) refers to physical activity characterized by relatively brief bursts of vigorous activity (85–100% of VO_2peak_), interspersed with short periods of rest or low-intensity physical activity to allow recovery (19, 20). HIIT requires less time and yields benefits similar to MICT (21). HIIT is better than MICT for improving ventilation (22) in obese patients, and MICT can result in fatigue and respiratory restriction (23). Some studies showed that, compared with MICT, HIIT has good efficacy in improving motor performance, cardiovascular function and reducing cardiovascular risk factors in patients with CVD (3, 24, 25). However, other studies have shown that both HIIT and MICT can improve cardiorespiratory fitness in patients with CVD (26–28). This controversy might be attributed to different training models, frequencies, and intervention durations in the different studies, complicating interpretation of results and clinical applications (29).

HIIT has been divided into three models defined by exercise and recovery times. Long-interval HIIT involves 4 min of high-intensity exercise interspersed with 3 min of active or passive recovery. Medium-interval HIIT involves 1–2 min of high-intensity exercise interspersed with 1–4 min of low-intensity recovery. Short-interval HIIT involves 15–60 s of high-intensity training interspersed with 15–120 s of low-intensity recovery (22, 30). However, which model of HIIT is most effective in improving cardiorespiratory fitness in patients with CVD, and how the various models compare with MICT, remains unclear (22).

Some studies have shown that HIIT twice a week, and even at lower frequencies, can significantly improve cardiorespiratory fitness (31, 32). Chin et al. found that HIIT once a week can improve cardiorespiratory fitness compared with no intervention, and HIIT 2–3 times a week can improve cardiorespiratory fitness to a greater extent than MICT (33). However, the American College of Sports Medicine (ACSM) guidelines state that only moderate to high-intensity continuous training or intermittent training at least three times a week can effectively improve cardiorespiratory fitness, while training <2 times a week will not yield significant improvement in healthy adults (34). Stavrinou et al. reported that HIIT twice weekly increases VO_2peak_ by 10.8%, while training three times a week increases VO_2peak_ by 13.6% (35). It has been reported that there is a dose-response relationship between lactate threshold and the frequency of intermittent training (36). Considering the physical condition of CVD patients, it is important to explore an optimal frequency of HIIT in CR.

It has been shown that intervention duration is a key factor determining adaptive changes in body function and structure in response to exercise (37). A previous systematic review and meta-analysis reported that more than 6 weeks of HIIT was superior to MICT in improving cardiorespiratory fitness in patients with CVD, and 7–12 weeks of HIIT was the largest improvements in cardiorespiratory fitness (3). However, some studies have yielded contradictory results (38, 39). For these reasons, this systematic review and meta-analysis of randomized controlled trials (RCTs) aimed to explore the effects of MICT and different HIIT training models and intervention frequencies and durations on cardiorespiratory fitness in patients with CVD.

## Materials and Methods

This systematic review and meta-analysis was carried out in conformance with PRISMA guidelines (40). The literature search and screening plan were pre-established. The protocol for this systematic review has been registered on PROSPERO (CRD42021245810).

### Literature Search

Articles were systematically searched journals indexed in the PubMed, Web of Science, Cochrane Library, Embase and Scopus databases from inception to December 2021 using the following terms: [(High-intensity interval training) OR (High-intensity interval exercise) OR (High-Intensity Intermittent Exercise) OR (Sprint Interval Training) OR (High-Intensity Intermittent Exercises) OR (Anaerobic interval exercise) OR (Exercise, High-Intensity Intermittent) OR (HIIT) OR (HIT) OR (HIIE)] AND [(Cardiac rehabilitation) OR (Rehabilitation, Cardiac) OR (Cardiovascular Rehabilitation) OR (Rehabilitation, Cardiovascular)]. We also searched the literature in other ways, retrieving gray literature, printed materials in the library, and references cited in the articles.

### Study Selection

Two researchers selected articles in an unblinded manner. When there were differences in their selections, a third researcher participated in the discussion to reach a final decision. Inclusion criteria for this systematic review and meta-analysis included (1) randomized controlled trials written in English; (2) adult patients with CVD who had undergone cardiac rehabilitation; (3) HIIT and MICT exercise interventions, but not other training (e.g., HIIT combined with strength training, intervention based on aquatic HIIT programs, etc.); (4) a clear statement of the type, intensity, duration, intervention time, frequency, and interval of the exercise intervention; (5) VO_2peak_ among the outcome measures; and (6) complete datasets with a report of the mean and standard deviation of VO_2peak_ before and after the intervention.

Exclusion criteria included (1) duplicated articles; (2) abstract and conference articles; (3) outcome measures without VO_2peak_; (4) incomplete reports of study data.

### Data Extraction

Two researchers independently read the full text of the literature in an unblinded manner and extracted outcomes. When there was disagreement, a third person participated in the discussion to reach a final decision. The extracted information included (1) citation (author and year of publication); (2) patient characteristics (sample size, age, gender and diagnosis); (3) intervention (exercise intervention type, duration, intensity and frequency); (4) outcome measures (pre- and post-VO_2peak_ values and changes of VO_2peak_); (5) adverse events.

### Study Quality

Study quality was assessed using the Cochrane Collaboration's tool (41) and the Physiotherapy Evidence Database (PEDro) Scale (42). Items of the Cochrane Collaboration's tool were evaluated in three categories: low risk of bias, unclear bias, and high risk of bias. The following characteristics were evaluated: random sequence generation (selection bias), allocation concealment (selection bias), blinding of participants and personnel (performance bias), incomplete outcome data (attrition bias), selective reporting (reporting bias), and other biases. The PEDro-scale included the following 11 items: eligibility criteria and source, random allocation, concealed allocation, baseline comparability, blinding of participants, blinding of therapists, blinding of assessors, adequate follow-up (>85%), intention-to-treat analysis, between-group statistical comparisons, reporting of point measures, and measures of variability (42). Eligibility criteria and source affected the external validity of the experiment without affecting internal and statistical validity; this item was therefore not used to calculate the PEDro score (42). The item “blinding of participants and blinding of therapists” did not apply to the intervention studies in CR (3). We removed these two items from the quality assessment, yielding a total score of eight.

### Statistical Analysis

Consistent with the purpose of this study, previous studies were collated according to the HIIT model (long-, medium-, or short-interval) (22, 30), HIIT intervention frequency (two, three, or five times a week) (43), and intervention duration (up to 6 weeks, 7–12 weeks, and more than 12 weeks) (3). The primary outcome was changes in VO_2peak_ after intervention in CR. The secondary outcome was adverse events, including cardiovascular events among others. An adverse event was defined as an event that occurred during or up to 4 h after an intervention session (44).

Pooled-effect estimates were obtained from the random-effects model and the mean differences (MDs) of the pre- to post-intervention values, from which the corresponding 95% confidence intervals (95% CI) were calculated. If studies did not provide the standard deviation (SD) of change in VO_2peak_, it was calculated using a correlation coefficient (r) of 0.5 and the following equation from the Cochrane Handbook (45):


(1) $$\begin{array}{r} SD_{change}=\sqrt{SD_{pre}^{2}+SD_{post}^{2}-\left(2r\times SD_{pre}\times SD_{post}\right)} \end{array}$$

Heterogeneity was assessed by Cochrane's Q and *I*^2^ static. *I*^2^ <25% indicates no significant heterogeneity; 25% < *I*^2^ <50%, low heterogeneity; 50% < *I*^2^ <75%, medium heterogeneity; *I*^2^ > 75%, high heterogeneity. Sensitivity analysis was used to examine the possible effects of individual studies on heterogeneity and overall effect of an intervention. This systematic review and meta-analysis was conducted using Review Manager 5.4 and Stata. The threshold for statistical significance was *p* < 0.05.

Publication bias was assessed with a visual inspection of funnel plots. Additionally, funnel plot asymmetry was statistically tested by Egger's test and *p* < 0.05 was considered significant (46). If there was any publication bias, the stability of the results was evaluated using a trim and fill method (47).

## Results

### Literature Search

**Figure 1:**
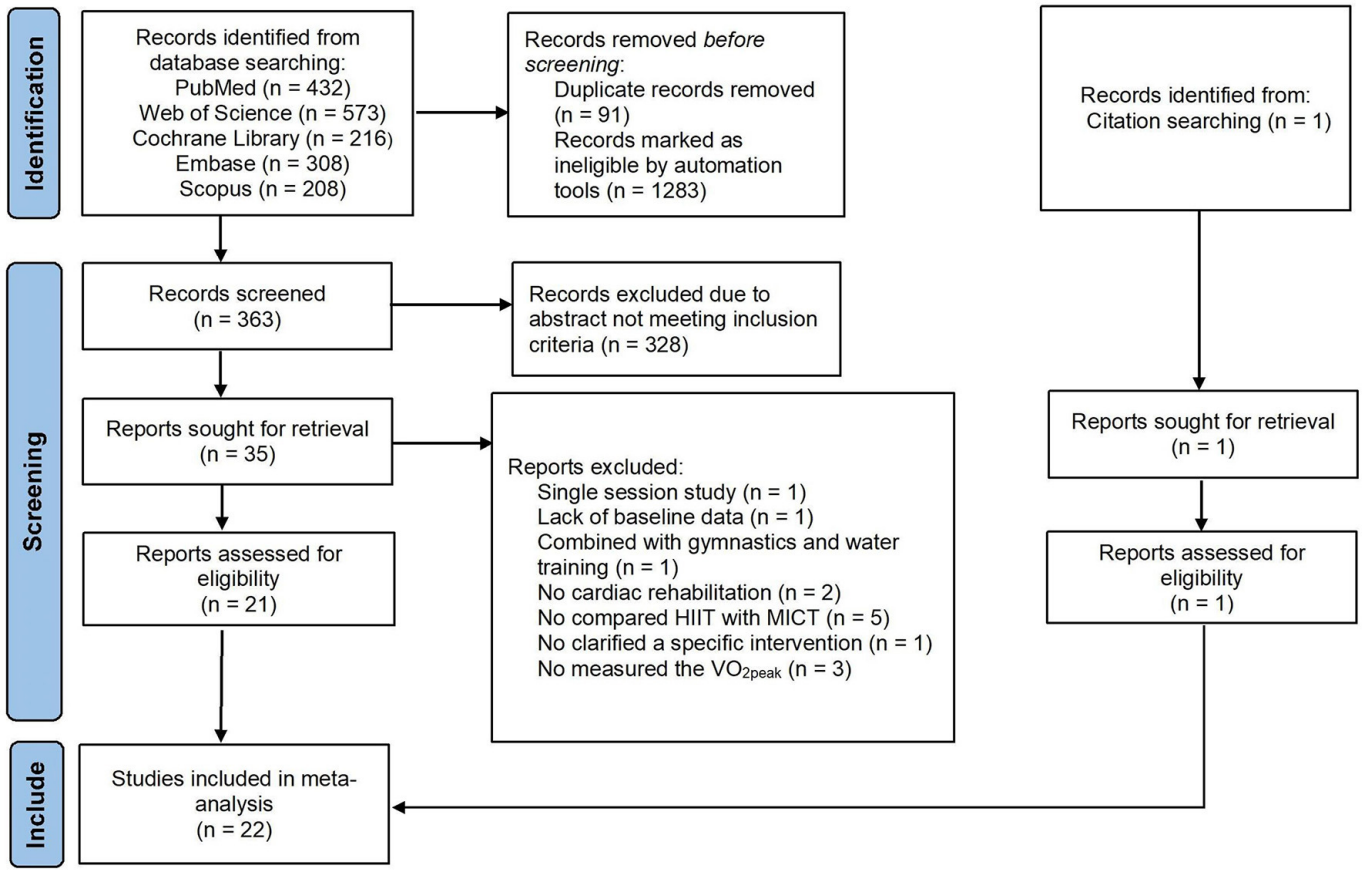
PRISMA flow diagram of literature search strategies. HIIT, high-intensity interval training; MICT, moderate-intensity interval training; VO_2peak_, peak oxygen uptake.

A PRISMA diagram of literature search and selection was presented in Figure 1. The initial search resulted in 1,738 articles from journals indexed in the PubMed, Web of Science, Cochrane Library, Embase, Scopus and other ways. The duplicated (*n* = 91) and ineligible documents (*n* = 1283) were excluded by automation tools. The remaining articles (*n* = 364) were screened. Three hundred and twenty-eight articles did not meet the inclusion criteria and thus were excluded. The remaining articles (*n* = 36) were read in full text and 22 articles were finally included in this study. Fourteen articles were excluded because of single-session intervention (*n* = 1), the lack of baseline data (*n* = 1), the study combined with gymnastics and underwater sports (*n* = 1), no cardiac rehabilitation (*n* = 2), without compared HIIT with MICT (*n* = 5), no clarified a specific intervention (*n* = 1) and no measured the VO_2peak_ (*n* = 3).

### Study Characteristics

The studies of RCTs were included from 2004 to 2020. There were 949 participants (age: 48 to 76 years), of which 476 participants were in the HIIT group, and 473 participants were in the MICT group. Not all studies reported gender, for those who did, 735 men and 155 women were reported. The studies reported the patients with coronary artery disease (25, 27, 48–56), heart failure (HF) (19, 28, 57–62), myocardial infarction (63, 64), and heart transplant patients (65) in CR.

In included studies, the intervention program included cycle ergometers and treadmill exercise except one study used a combination of a stair climber, treadmill, and arm/leg ergometer exercise (49). The HIIT models included short-interval training model in six studies (25, 50, 54, 57–59), medium-interval training model in two studies (49, 52), and long-interval training model in 14 studies (19, 27, 28, 48, 51, 53, 55, 56, 60–65). All studies based on maximum/peak test data to set exercise intensity, such as VO_2peak_, HR_peak_ (peak heart rate), VO_2_R (oxygen uptake reservation), HRR (heart rate reservation), PPO (peak power output), maximum workload, maximum effort, and respiratory compensation point. Intervention duration was from 3.5 weeks to 9 months, with five studies reporting for 0–6 weeks (55, 56, 59, 60, 63), 15 studies reporting for 7–12 weeks (19, 25, 27, 28, 48, 50, 51, 53, 54, 56–58, 61, 62, 64), and four studies reporting data more than 12 weeks (49, 52, 57, 65). The intervention frequency was between 2 and 5 times per week, with 16 studies for three times per week (19, 25, 27, 48, 51–57, 60–63, 65), three studies for two times per week (49, 50, 58), one study for five times per week (59) and two study performed dynamic frequency (28, 64). The duration of intervention sessions ranged from 25 to 50 min. Seventeen studies were supervised by professional therapists and five studies were unsupervised. The monitor control index incorporated the heart rate, blood pressure, electrocardiogram, and RPE (rating of perceived exertion). Descriptive characteristics of the included studies were shown in Table 1.

**Table 1:** Descriptive characteristics of the included studies.

**Study**	**Participants**			**Duration, and frequency**	**Exercise intervention**	
	**Age**	**Gender (M/F)**	**Population**		**HIIT**	**MICT**
Rognmo et al. (48)	HIIT 62.9 ± 11.2 MICT 61.2 ± 7.3	HIIT 6/2 MICT 8/1	CAD	10 wks; 3 times / wk	4*4-min intervals at 85–95% HR_peak_, interspersed by 3 min active recovery at 65–75% HR_peak_	41 min at 65–75% HR_peak_
Warburton et al. (49)	HIIT 55 ± 7 MICT 57 ± 8	HIIT 7/0 MICT 7/0	CAD	16 wks; 2 times / wk	2 min at 85–95% HRR/VO_2_R interspersed by 2 min active recovery at 35–45% HRR/VO_2_R, a total of 30 min	30 min at 60% HRR/VO_2_R
Wisløff et al. (19)	HITT 76.5 ± 9 MICT 74.4 ± 12	HIIT 7/2 MICT 7/2	HF	12 wks; 3 times / wk	4*4-min intervals at 90–95% HR_peak_, interspersed by 3 min active recovery at 50–70 % HR_peak_	47 min at 70–75% HR_peak_
Iellamo et al. (28)	HIIT 62.2 ± 8 MICT 62.6 ± 9	HIIT 8/0 MICT 8/0	HF with reduced ejection fraction	12 wks; 2–5 times / wk	4*4-min intervals at 75–80% HRR, interspersed by 3 min active recovery at 45–50% HRR	30–45 min at 45–60% HRR
Currie et al. (50)	HIIT 62 ± 11 MICT 68 ± 8	HIIT 11 MICT 11 Total 20/2	CAD	12 wks; 2 times / wk	10*1-min intervals at 80–104 % PPO, interspersed by 1 min active recovery at 10% PPO	30–50 min at 51–65% PPO
Keteyian et al. (51)	HIIT 60 ± 7 MICT 58 ± 9	HIIT 11/4 MICT 12/1	CAD	10 wks; 3 times / wk	4*4-min intervals at 80–90% HRR, interspersed by 3 min active recovery at 60–70% HRR	30 min at 60–80% HRR
Koufaki et al. (57)	Total:59.1 ± 8.6	HIIT 8 MICT 9 Total 14/3	HF with reduced ejection fraction	12 wks; 3 times / wk	2*15 min bouts,30 s at 50% of the maximum workload reached with the MSEC test (100% PPO), interspersed by 1 min recovery periods at 20–30% of peak power output (25–40 watts)	40 min at 40–60% VO_2peak_
Koufaki et al. (57)	Total:59.1 ± 8.6	HIIT 8 MICT 9 Total 14/3	HF with reduced ejection fraction	24 wks; 3 times / wk	2*15 min bouts,30 s at 50% of the maximum workload reached with the MSEC test, interspersed by 1 min recovery periods at 20–30% of peak power output (25–40 watts)	40 min at 40-60% VO_2peak_
Angadi et al. (60)	HIIT 69.0 ± 6.1 MICT 71.5 ± 11.7	HIIT 8/1 MICT 4/2	HF with preserved ejection fraction	4 wks; 3 times / wk	4*4-min intervals at 85–90% HR_peak_, interspersed by 3 min active recovery at 50% HR_peak_	30 min at 70% HR_peak_
Kim et al. (63)	HIIT 57 ± 11.58 MICT 60.2 ± 13.64	HIIT 12/2 MICT 10/4	Acute myocardial infarction patients with drug-eluting stent	6 wks; 3 times / wk	4*4-min intervals at 85–95% HRR, interspersed by 3 min active recovery at 50–70% HRR	25 min at 70–85% HRR
Benda et al. (58)	HIIT 63 ± 8 MICT 64 ± 8	HIIT 9/1 MICT 10/0	HF with reduced ejection fraction	12 wks; 2 times / wk	10*1-min intervals at 60–75% of maximal workload and Borg score of 15–17, interspersed by 2.5 min active recovery at 30% of maximal workload	30-min at 60–75% of maximal workload, Borg score of 12–14
Cardozo et al. (52)	HIIT 56 ± 12 MICT 62 ± 12	HIIT 14/9 MICT 16/8	CAD	16 wks; 3 times / wk	2 min at 90% HR_peak_, interspersed by 2 min active recovery at 60% HR_peak_, a total of 30 min	30 min at 70–75% HR_peak_
Jaureguizar et al. (25)	HIIT 58 ± 11 MICT 58 ± 11	HIIT 28/8 MICT 33/3	CAD	8 wks; 3 times / wk	In the first month, 20 s at 50% of the maximum load reached with the SRT, interspersed by 40 s recovery periods at 10% of the maximum load, the total duration was 40 min. In the second month, the intensity of exercise was adjusted using the results of a new SRT	40 min below the HR at VT_1_ during the first month. During the second month, the intensity of the exercise was adjusted, increasing to a training HR that corresponded to VT_1_ plus 10%
Prado et al. (53)	HIIT 56.5 ± 2.7 MICT 61.3 ± 2.2	HIIT 14/3 MICT 14/4	CAD	12 wks; 3 times / wk	7*3-min intervals at the respiratory compensation point, interspersed by 3 min active recovery at VAT intensity	50 min at VAT intensity.
Conraads et al. (56)	HIIT 57.8 ± 8.8 MICT 59.9 ± 9.2	HIIT 91/9 MICT 89/11	CAD	6 wks; 3 times / wk	4*4-min intervals at 85–95% HR_peak_, interspersed by 3 min active recovery at 50–70% HR_peak_	37 min at 70–75% HR_peak_
Conraads et al. (56)	HIIT 57.8 ± 8.8 MICT 59.9 ± 9.2	HIIT 91/9 MICT 89/11	CAD	12 wks; 3 times / wk	4*4-min intervals at 85–95% HR_peak_, interspersed by 3 min active recovery at 50–70% HR_peak_	37 min at 70–75% HR_peak_
Besnier et al. (59)	HIIT 59 ± 13 MICT 59.5 ± 12	HIIT 11/5 MICT 11/4	HF with reduced ejection fraction	3.5 wks; 5 times / wk	2*8 min blocks, 30 s at 100% peak power output, interspersed by 30 s passive recovery	30 min at 60% peak power output
Jaureguizar et al. (54)	HIIT 57.6 ± 9.8 MICT 58.3 ± 9.5	HIIT 50/7 MICT 42/11	CAD	8 wks; 3 times / wk	In the first month, 20 s at 50% of the maximum load reached with the SRT, interspersed by 40 s recovery periods at 10% of the maximum load, the total duration was 40 min. In the second month, the intensity of exercise was adjusted using the results of a new SRT	40 min below the HR at VT_1_ during the first month. During the second month, the intensity of the exercise was adjusted, increasing to a training HR that corresponded to VT_1_ plus 10%
Rolid et al. (65)	HIIT 50 ± 12 MICT 48 ± 14	HIIT 28/9 MICT 29/12	Heart transplantation	36 wks; 3 times / wk	4*4-min intervals at 85–95% maximal effort (RPE 16–18), interspersed by 3 min active recovery at RPE 11-13	25 min at 60–80% maximal effort (RPE 12–15)
Choi et al. (64)	HIIT 53.00 ± 6.84 MICT 57.31 ± 12.62	HIIT 21/2 MICT 18/3	MI	9-10 wks; 1-2 times / wk	4*4-min intervals at 85–100% HR_max_, interspersed by 3 min active recovery at 50–60% HR_max_	28 min at 60–70% HR_max_
Anderson et al. (61)	HIIT 60 ± 10 MICT 60 ± 9	HIIT 3/7 MICT 4/5	HF with preserved ejection fraction	12 wks; 3 times / wk	4*4-min intervals at 85–95% HR_peak_, interspersed by 3 min active recovery at 60–70% HR_peak_	47 min at 60–70% HR_peak_
Rocco et al. (27)	HIIT 56.5 ± 3.0 MICT 62.5 ± 2.0	HIIT 14/3 MICT 15/5	CAD	12 wks; 3 times / wk	7*3-min intervals at the respiratory compensation point, interspersed by 3 min active recovery at VAT intensity	50 min at VAT intensity
Ulbrich et al. (62)	HIIT 53.15 ± 7.0 MICT 54.02 ± 9.9	HIIT 12/0 MICT 10/0	HF	12 wks; 3 times / wk	3 min at 95% HR_peak_, interspersed by 3 min active recovery at 70% HR_peak_, a total of 40 min	40 min at 75% HR_peak_
Taylor et al. (55)	HIIT 65 ± 7 MICT 65 ± 8	HIIT 43 MICT 43 Total 86	CAD	4 wks; 3 times / wk	4*4-min intervals at 15–18 RPE, interspersed by 3 min active recovery at 11–13 RPE	40 min at 11–13 RPE

*M, male; F, female; HR, heart rate; HR_peak_, peak heart rate; HRR, heart rate reservation; VO_2_R, oxygen uptake reservation; VO_2peak_, peak oxygen uptake; PPO, peak power output; MSEC, maximum short exercise capacity; SRT, steep ramp test; VAT, ventilatory anaerobic threshold; VT_1_, the first ventilatory thresholds; RPE, rating of perceived exertion, Wks; weeks; CAD, coronary artery disease; HF, heart failure; MI, Myocardial Infarction*.

### Quality Assessment

**Figure 2:**
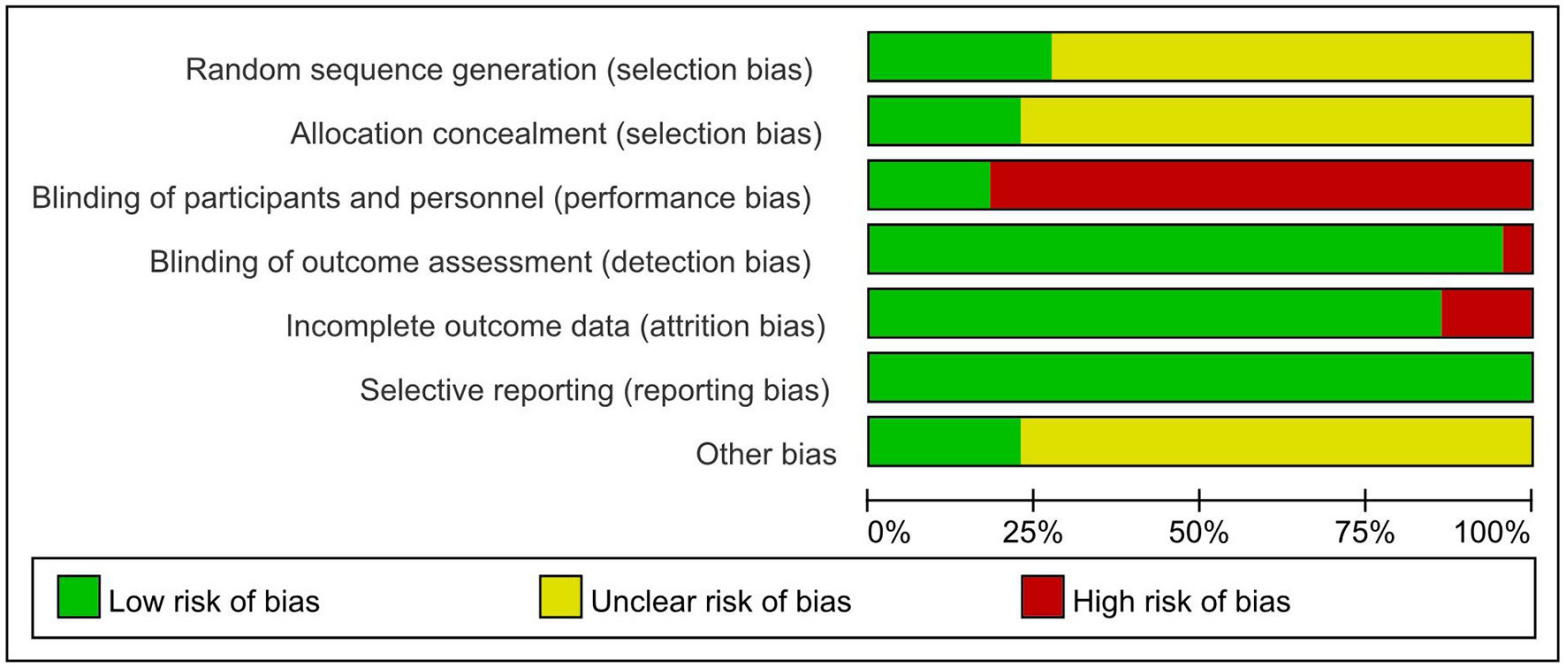
Summary of risk of bias by domain.

Two researchers independently assessed the quality of the included studies and discrepancies were resolved by consensus. The quality of the included studies was evaluated using the Cochrane Collaboration's tool and the result showed reasonably (Figure 2). The quality of rehabilitation trials was assessed by the PEDro scale and the score ranged from 4 to 7.

### Sensitivity Analysis

The total heterogeneity and the subgroup heterogeneity for long-interval HIIT, three times a week and 7–12 weeks were 13, 28, 22, and 35%, respectively. To verify the reliability of the findings, we excluded the literature one by one and examined whether each article had a significant effect on the pooled results. Sensitivity analysis showed that the study of Wisløff et al. had a significant effect on the combined results of this meta-analysis (19). After removing this study, the total heterogeneity and intra subgroup heterogeneity of this meta-analysis dropped to 0%.

In the Wisløff et al. study, the participants were mainly diagnosed with heart failure and cardiac dysfunction (mean left ventricular ejection fraction 29%), and the baseline VO_2peak_ was very low (19). This might be the reason for the large heterogeneity. Therefore, we excluded this literature and performed a meta-analysis of the remaining 21 articles (23 studies).

### Changes of VO_2peak_: Meta-Analysis Results

**Figure 3:**
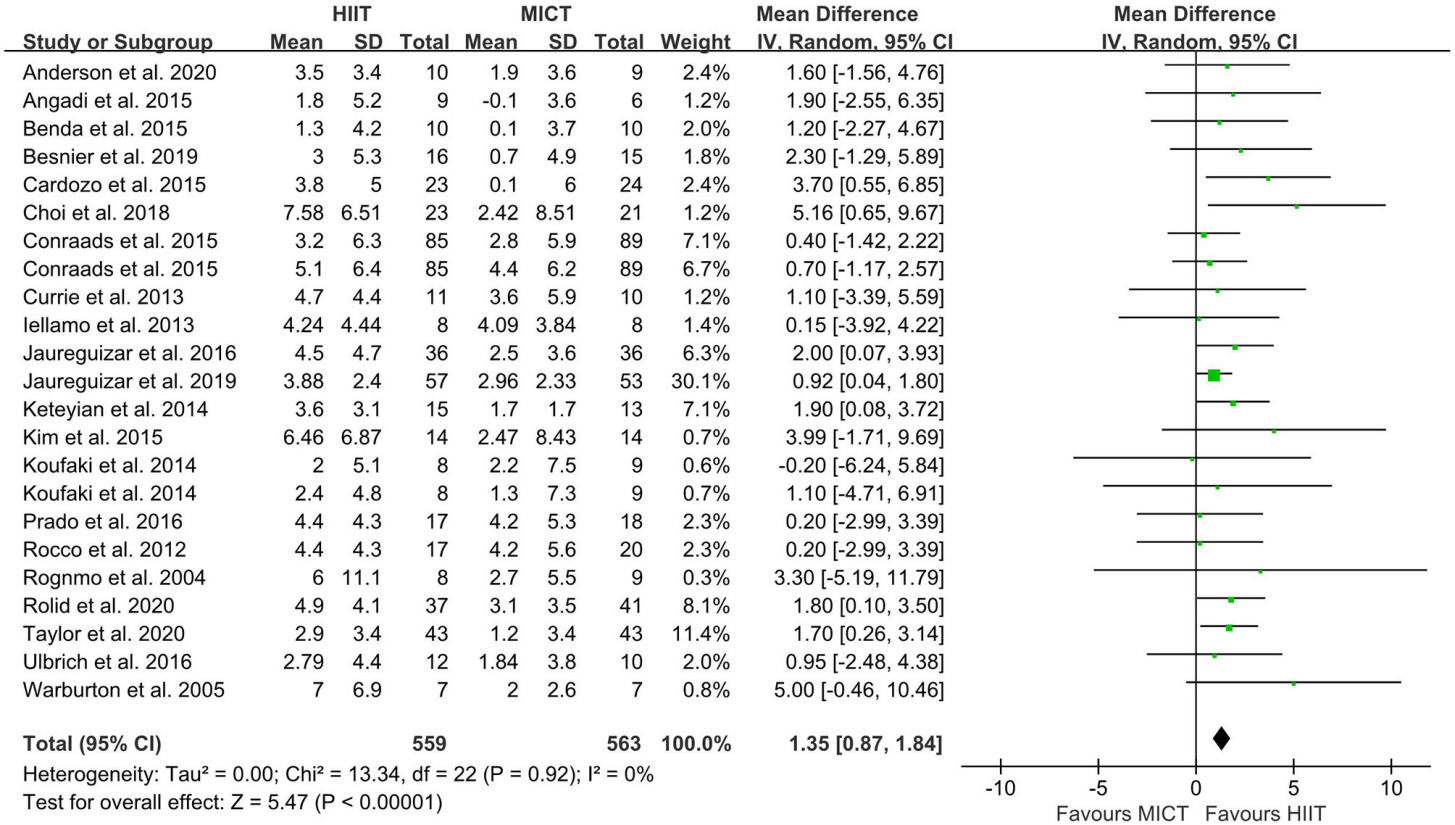
Forest plot depicting cardiorespiratory fitness changes as a HIIT vs. MICT. HIIT, high-intensity interval training; MICT, moderate-intensity continuous training.

**Figure 4:**
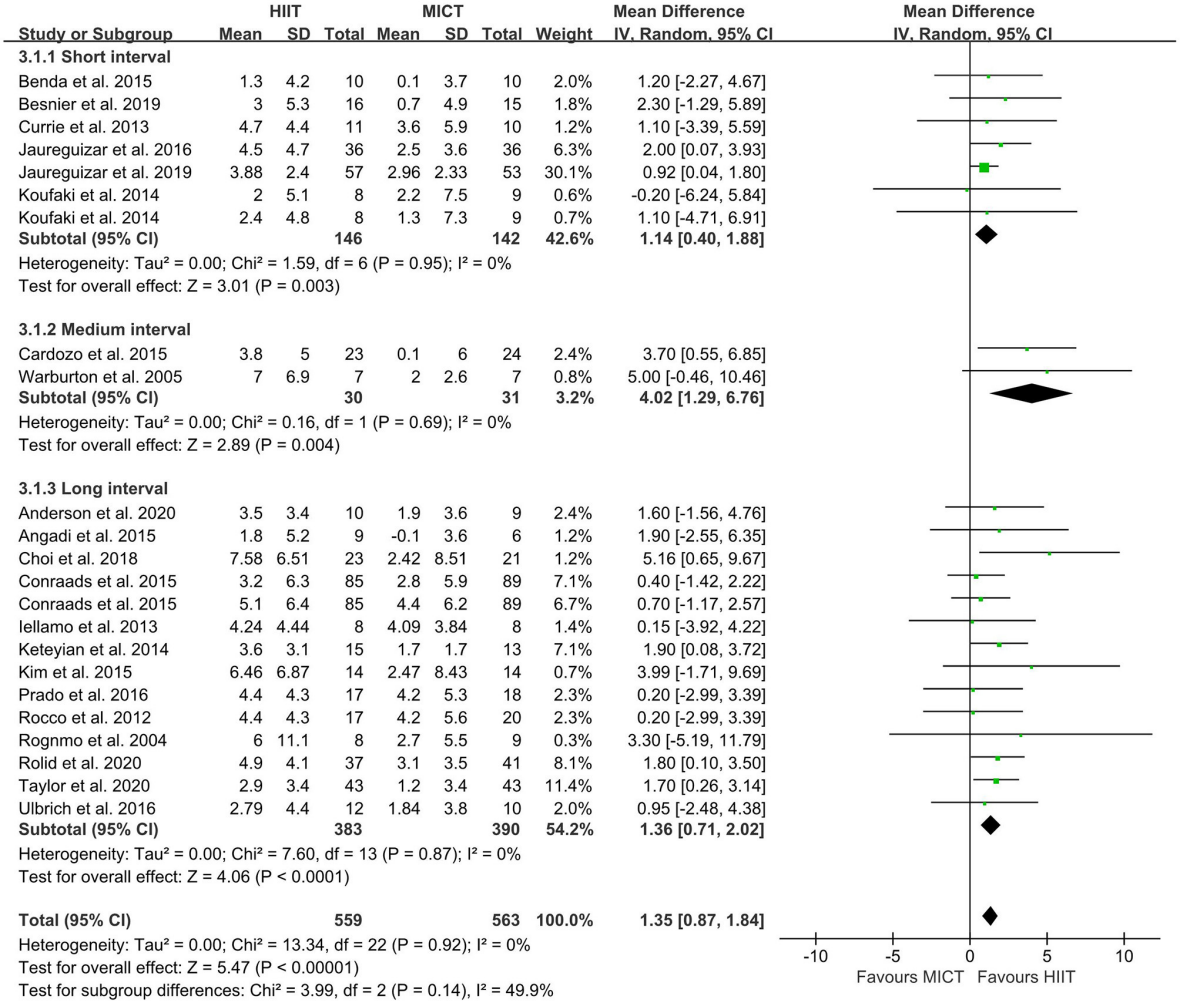
Forest plot of subgroup analysis by a different model of HIIT (short-interval, medium-interval and long-interval HIIT). HIIT, high-intensity interval training; MICT, moderate-intensity continuous training.

**Figure 5:**
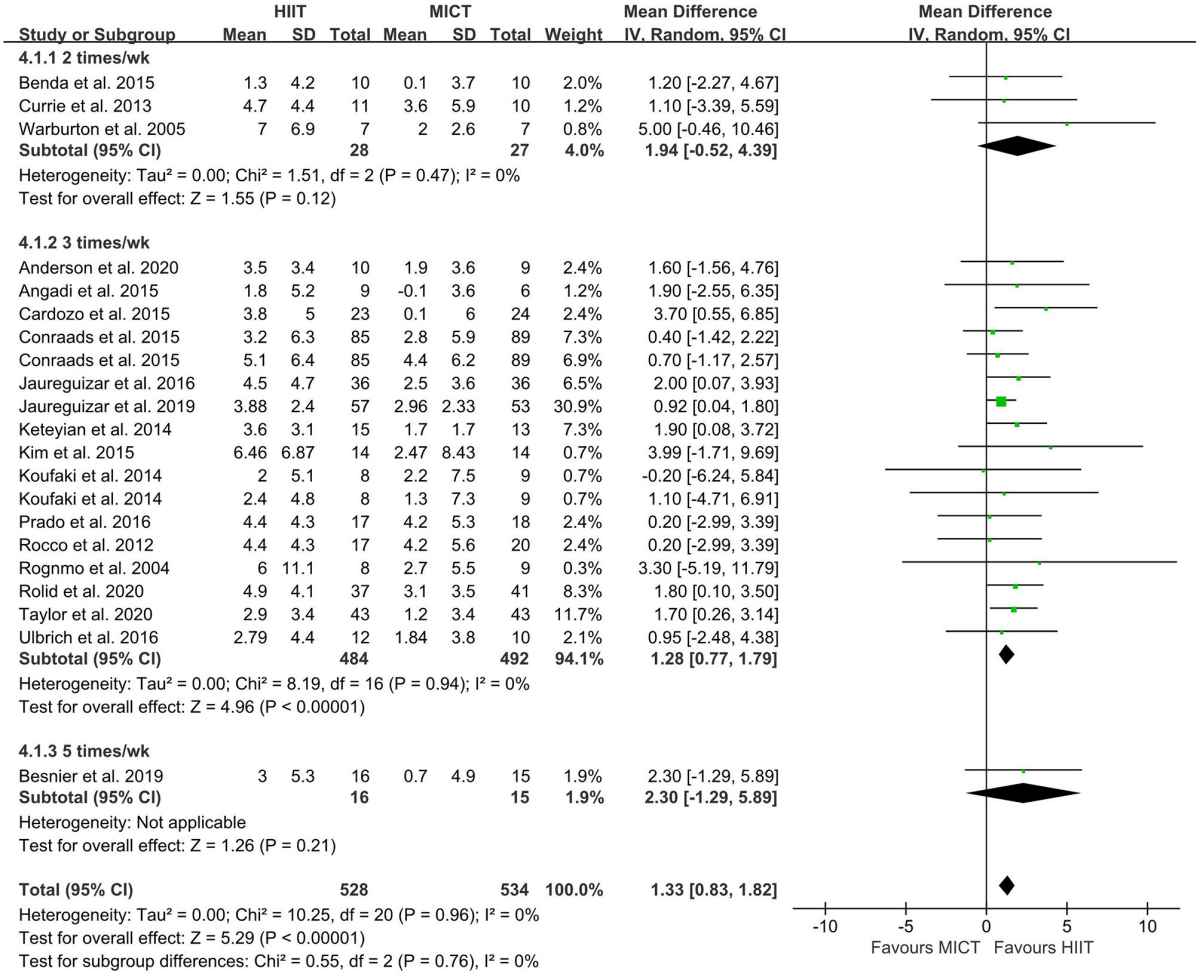
Forest plot of subgroup analysis by different frequencies of HIIT (2 times a week, 3 times a week, and 5 times a week). HIIT, high-intensity interval training; MICT, moderate-intensity training.

**Figure 6:**
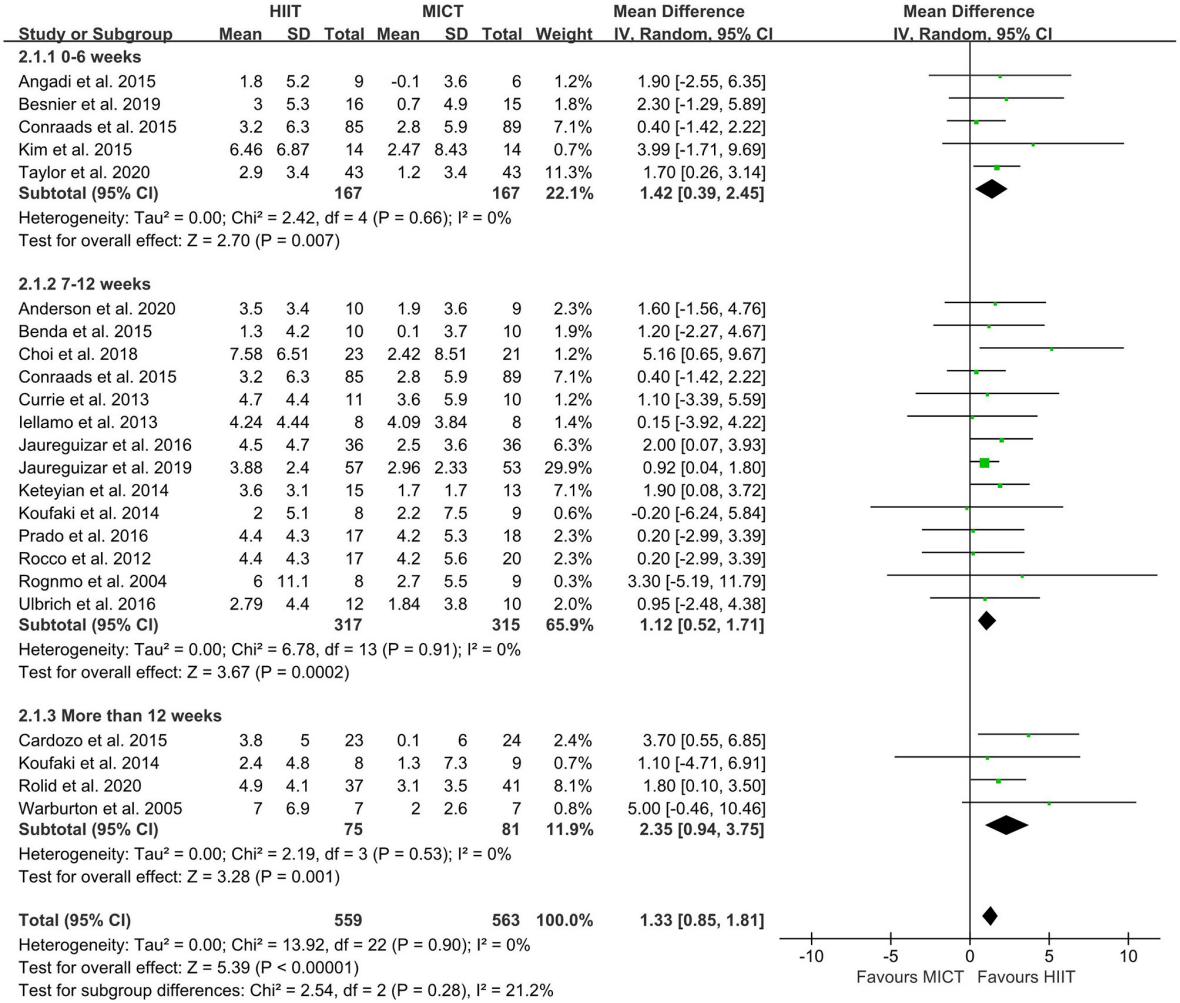
Forest plot of subgroup analysis by different intervention duration (0–6 weeks, 7–12 weeks, more than 12 weeks). HIIT, high-intensity interval training; MICT, moderate-intensity training.

The random-effect model showed that VO_2peak_ of patients with CVD was significant improvement in HIIT group as compared with MICT group (MD = 1.35, 95% CI = 0.87–1.84, *I*^2^ = 0%, *p* < 0.00001, Figure 3). In HIIT model, VO_2peak_ was significant increasement in short-interval HIIT (MD = 1.14, 95% CI = 0.40–1.88, *I*^2^ = 0%, *p* = 0.003), medium-interval HIIT (MD = 4.02, 95% CI = 1.29–6.76, *I*^2^ = 0%, *p* = 0.004) and long-interval HIIT (MD = 1.36, 95% CI = 0.71–2.02, *I*^2^ = 0%, *p* < 0.0001) in comparison with MICT group (see Figure 4). In intervention frequencies of HIIT, there was a significant improvement in VO_2peak_ using HIIT three times a week (MD = 1.28, 95% CI = 0.77–1.79, *I*^2^ = 0%, *p* < 0.00001, Figure 5). VO_2peak_ showed a significant improvement in HIIT group with 0–6 weeks (MD = 1.42, 95% CI = 0.39–2.45, *I*^2^ = 0%, *p* = 0.007), 7–12 weeks (MD = 1.12, 95% CI = 0.52–1.71, *I*^2^ = 0%, *p* = 0.0002) and >12 weeks (MD = 2.35, 95% CI = 0.94–3.75, *I*^2^ = 0%, *p* = 0.001) as compared with MICT group (see Figure 6).

### Adverse Events

Adverse events related to exercise intervention were reported for 17 of 21 studies (80.95%). Eleven adverse events were reported. There was only one minor cardiovascular event in the HIIT group and the patient had syncope during one session, but continued to participate in the study. The other ten adverse events were classified as non-cardiovascular. Four adverse events occurred in the HIIT group: knee pain, ankle injury and ankle fracture. The other six adverse events were in the MICT group: leg pain, knee injury, anxiety/panic attack, back pain, epilepsy, knee pain (prosthesis) and ankle injury.

### Publication Bias

**Figure 7:**
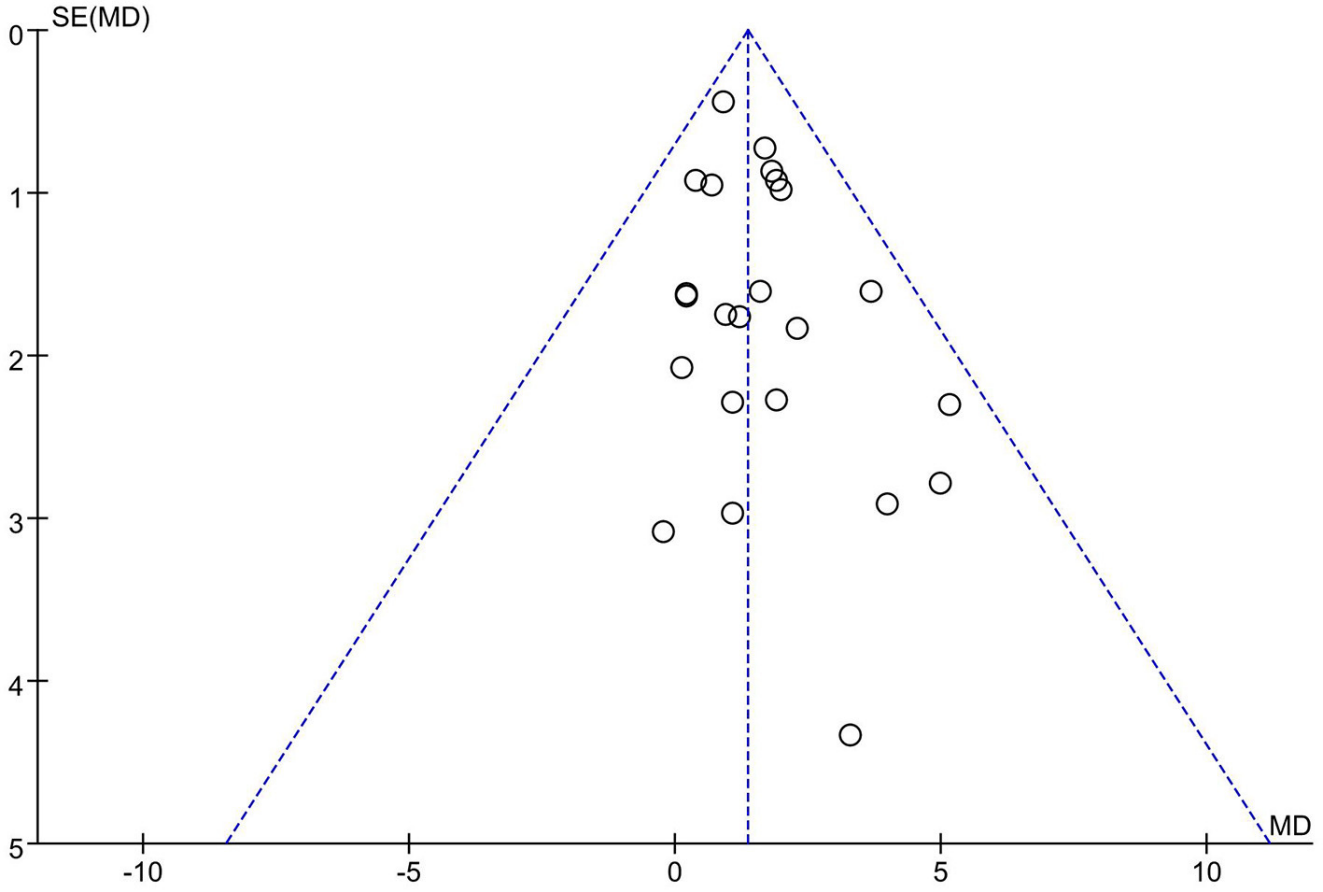
Funnel plot of publication bias.

**Figure 8:**
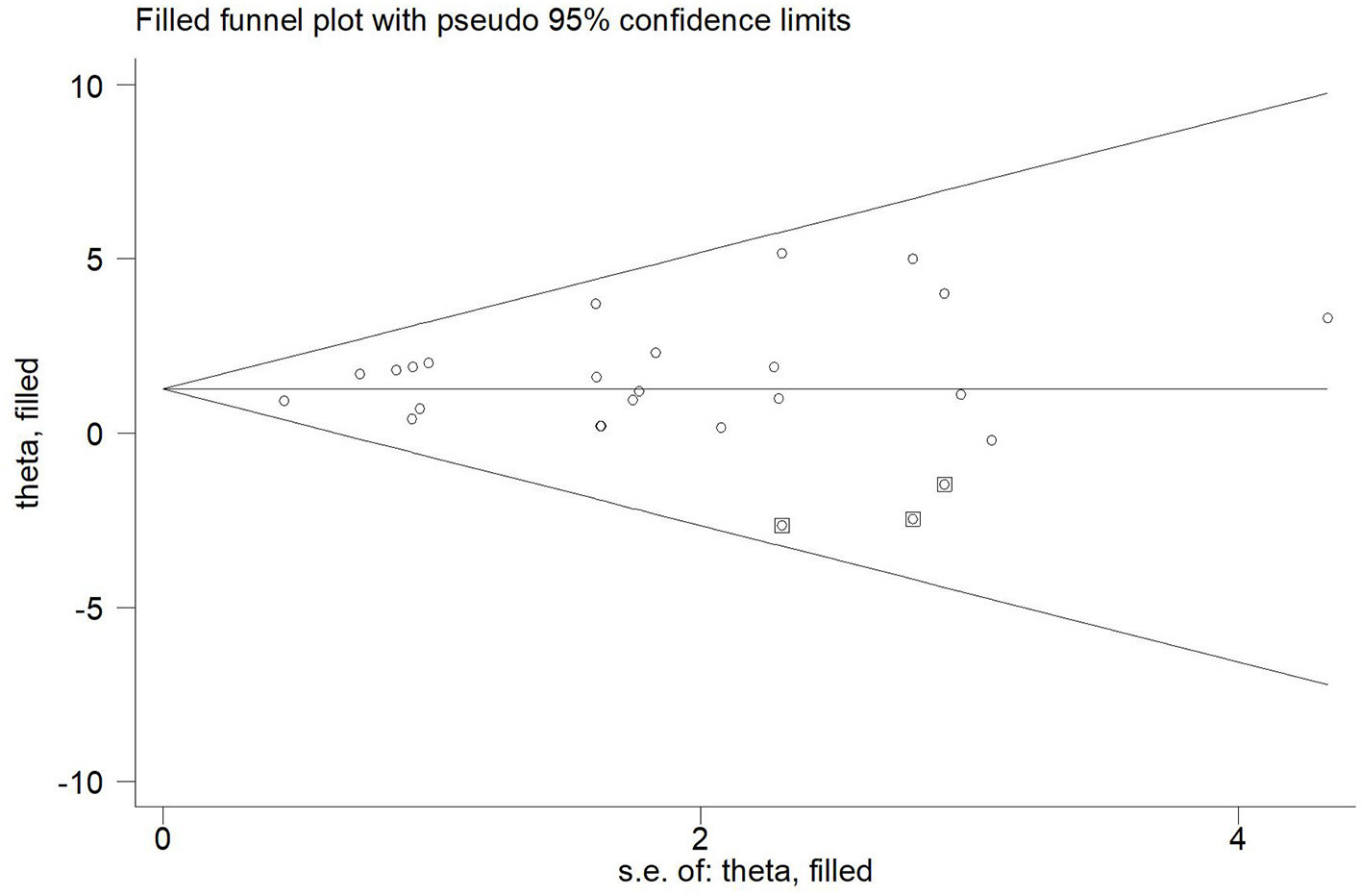
The funnel plot showed the trim and fill method adjusted publication bias. ○, previous studies; 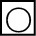 filled studies.

Twenty-one articles (23 studies) were examined for publication bias. Visual inspection of the funnel plot (Figure 7) was asymmetry, but Egger's test (*p* = 0.101) revealed there was no significant publication bias. The trim and fill adjusted 26 studies, and the mean difference was 1.26 (95% CI = 0.78–1.74). The three imputed hypothetical studies produced a symmetrical funnel plot (Figure 8). Further research would include the three studies to guarantee the symmetry of the funnel chart and eliminate potential publication bias.

## Discussion

This systematic review and meta-analysis carried out here identified different HIIT models for improving VO_2peak_ in patients with CVD, and explored the most effective intervention frequency and duration to optimize HIIT. In contrast to previous meta-analyses (3, 43, 66), our study included new and large-sample trials as well as multicenter randomized controlled trials. To our knowledge, this is the first study to explore which model of HIIT provides the greatest benefits for cardiorespiratory fitness in CR when compared with MICT. The results revealed that HIIT is superior to MICT for improving cardiorespiratory fitness in patients with CVD. Medium-interval HIIT 3 times/week for more than 12 weeks resulted in the greatest improvement in cardiorespiratory fitness in CR.

The meta-analysis in this study showed that HIIT increased VO_2peak_ much more than MICT. These results are consistent with the report of Liou et al. that HIIT improves VO_2peak_ in patients with coronary artery disease (CAD) (29). The meta-analysis of Pattyn et al. also showed that HIIT elicits larger increases in VO_2peak_ than does MICT in patients with CAD (67). Studies have shown that cardiorespiratory fitness is a strong predictor of cardiovascular disease and mortality (12). Compared with MICT, our meta-analysis showed that HIIT intervention elicited a 1.35 mL/kg/min greater improvement in VO_2peak_. This is of clinical significance because an increase in VO_2peak_ reduces the risk of all-cause mortality in patients with CAD and HF (68, 69).

The improvement in VO_2peak_ using HIIT occurred over periods of 0–6 weeks, 7–12 weeks and >12 weeks, with the maximum benefit observed at >12 weeks. Intervention duration plays an important role in the efficacy of HIIT (37). For patients with chronic heart failure, 16 weeks may be enough to achieve maximum improvement in function (> 15%), as suggested by a systematic review (70, 71). Moreover, unpublished data in the Smart and Steele review showed that VO_2peak_ increased by 13% after 8 weeks of aerobic exercise and 21% after 16 weeks (71). Moholdt et al. trained patients who had undergone coronary artery bypass grafting and found that VO_2peak_ was not significantly different in the HIIT and MICT groups at the fourth week, but was significantly higher in the HIIT group after 6 months (72). Jurio-iriarete and Maldonado-Mar-tin also reported that HIIT of <12 weeks did not improve cardiorespiratory fitness any more than MICT, but there did seem to be a greater increase with HIIT after 12 weeks (38). The study showed that long-term HIIT is significantly better than short-term HIIT or MICT in improving VO_2peak_ in overweight/obese adults with hypertension (38). Guadalupe-Grau et al. showed that up to 6 months of HIIT of middle-aged patients with metabolic syndrome not only improved skeletal muscle deoxygenation and oxygen extraction, but also increased mitochondrial enzyme activity and VO_2peak_ (73). Stroke volume, heart rate, cardiac output, and blood volume are core parameters that affect VO_2peak_ (30). A previous study showed that long-term HIIT is significantly superior to MICT in improving cardiac output and stroke volume in CR (74). Long-term HIIT can increase stroke volume (75) and improve cardiac autonomic function (76) *via* baroreflex-mediated augmentation of sinoatrial node regulation, enhancing VO_2peak_ as well as improving resting heart rate (67). Long-term HIIT resulted in greater adaptive changes in the musculoskeletal and cardiovascular systems in patients with CVD, and more than 12 weeks of HIIT was associated with a reduction in risk factors for CVD (38). The intensity-dependent improvements in the cardiovascular and musculoskeletal systems can account for HIIT being more effective than MICT in improving VO_2peak_ (67). The type of skeletal muscle, number of muscle fibers, density of capillaries, and content of mitochondria all contribute to uptake and utilization of oxygen (77). Moreover, HIIT can increase PGC-1 α and the body's oxidative capacity, as well as glucose uptake (19, 78). Long-term HIIT can increase the number and density of mitochondria and improve maximum metabolic capacity (79).

With respect to the HIIT models, the present meta-analysis showed that VO_2peak_ increased significantly in short-, medium- and long-interval HIIT, but the medium-interval model had the greatest effect. In a previous study, a multicenter RCT showed that long-interval HIIT reduced ejection fraction in patients with heart failure to a greater extent than did MICT (39). This indicated that the long-interval HIIT model was superior to MICT in improving cardiac remodeling and increasing cardiorespiratory fitness. However, this study found that only 51% of patients in the long-interval HIIT group maintained their target heart rate throughout the exercise. This implied that many geriatric patients with CVD were unable to perform prolonged high-intensity exercise. The average intensity (%VO_2peak_) of long-interval HIIT was higher, but there was lower tolerance and exercise compliance (22, 80, 81), which was presumably a result of long-interval HIIT being more burdensome than short- and medium-interval HIIT for patients with CVD (82). Conraads et al. found that the mean HR of patients with CAD did not reach the level required to sustain long-interval HIIT, and that training intensity had to be reduced for several patients to allow completion of the pedaling exercise or avoid extreme hyperventilation (56).

Patients in the long-interval HIIT group experienced more shortness of breath and had a higher Borg score than did those in the MICT group. Therefore, the study suggested that long-interval training at 90–95% of HR_peak_ was not feasible for most of the CAD patients. In contrast, Valstad et al. showed that short-interval training of healthy college students tended to lower lactate acid (LA) concentration as well as RPE and was perceived to be easier than long-interval training (83). Ballesta et al. (43) and Ribeiro et al. (84) demonstrated that short-interval HIIT is beneficial for CVD patient compliance with long-term treatment. Some studies reported that short-interval HIIT improved cardiorespiratory fitness in patients with CVD (25, 58, 59). Short-interval HIIT has a shorter exercise time and more training sets compared with the medium- and long-interval models. Although short-interval HIIT saves time and is similar in training efficacy to long-interval HIIT, 15–60 s of high-intensity training is too short for patients to reach the target intensity (82). This would imply that this model might be not sufficient to produce superior benefits (85, 86). Some studies have also shown that short-interval HIIT is not superior to MICT in patients with CVD (50, 71, 87). In our study, the ability of long-interval HIIT to improve cardiorespiratory fitness in patients with CVD was shown to be greater than that of short-interval HIIT, but medium-interval HIIT was superior to both. Similarly, Cardozo et al. showed that medium-interval HIIT was superior to MICT in improving cardiorespiratory fitness in patients with CAD (52). This implies that medium-interval HIIT is more suitable for persuading patients with CVD to maintain high intensity training and to achieve the target intensity because it involves relatively moderate exercise and interval times.

Regarding HIIT frequency, three times per week increased VO_2peak_. This result is consistent with the exercise frequency recommended by ACSM guidelines. One study used an intervention frequency of five times a week, so this result needs to be interpreted with caution. Similarly, Ballesta et al. in a meta-analysis of HIIT for patients with heart failure showed that HIIT three or four times a week has a significant effect on VO_2peak_, while no significant change was observed when two times a week was used (43). Kavaliauskas et al. found that sprint interval training (SIT) twice a week did not improve cardiorespiratory fitness for untrained young healthy women (88). The intensity of SIT was higher than that of HIIT, but the VO_2peak_ of participants did not improve. This implied that training frequency is an important variable in determining the physiological effects of SIT. Some studies have shown that HIIT once or twice a week improves cardiorespiratory fitness, but the participants were healthy adults or athletes and their baseline exercise capacity and health status were generally higher than those of patients with CVD. As suggested in the ACSM guidelines, HIIT at least three times a week can increase VO_2peak_ to achieve central and peripheral adaptive changes in CR. These studies indicated that HIIT three times a week might be the lowest training frequency sufficient to increase cardiorespiratory fitness in CR.

Our study found that one minor cardiovascular adverse event and four non-cardiovascular adverse events were reported in the HIIT group. Six non-cardiovascular adverse events were reported in the MICT group. Similarly, Wewege et al. (44) carried out a meta-analysis of 23 studies of CR (HIIT: 547 patients, MICT: 570 patients) and found one minor cardiovascular adverse event and three non-cardiovascular adverse events in the HIIT group and two non-cardiovascular events in the MICT group. A systematic review reported that no deaths or major cardiovascular events occurred in 17 studies of CR (HIIT: 465, MICT: 488) (3). Rognmo et al. (89) retrospectively analyzed cardiovascular adverse events in 4,846 patients with CAD and found that there was one case of fatal cardiac arrest per 129,456 patient-exercise hours for MICT and 1 per 23,182 h for HIIT. This indicated that both HIIT and MICT are at low risk of a cardiovascular event for patients with CAD in CR (89). The physical and rehabilitation medicine (PRM) physician is crucial in CR. The key responsibilities of PRM physicians are to develop and implement safe CR procedures (15) and to closely monitor patients during CR (90). Therefore, PRM physicians can help patients with CVD to reduce the incidence of adverse events.

## Strengths and Limitations

To our knowledge, this study included all literature prior to December 2021, and therefore has a large sample size. This is the first study of the effects of long-, medium- and short-interval HIIT on improvement of cardiorespiratory fitness in patients with CVD. The strengths of systematic reviews and meta-analyses include greater precision and statistical power of the estimates, but potential drawbacks include heterogeneity of the studies and publication bias (67). Imputed hypothetical studies accounted for potential publication bias in Figure 8, and the results are not meaningfully changed. Furthermore, the heterogeneity in similar earlier studies was large, while that of our study was low.

There were some limitations to this study. This study included many male participants, which may cause bias in the results. Only two studies in the medium-interval HIIT group were compared with MICT, and one study included HIIT five times a week, so the results from those meta-analyses have to be interpreted with some caution.

## Conclusion

This systematic review and meta-analysis found that HIIT is safe and appears superior to MICT for improving cardiorespiratory fitness in patients with CVD. To optimize these benefits, medium-interval HIIT three times/week for more than 12 weeks is recommended for improving cardiorespiratory fitness in patients with CVD.

## Future Directions

Future research should explore (1) the effects of medium-interval HIIT at least three times a week for more than 12 weeks in patients with CVD; (2) the long-term benefits of HIIT in patients with CVD and whether the exercise regiment is maintained. In addition, further research should recruit more female participants to examine whether HIIT is superior to MICT in a broader range of CVD patients in CR.

## Data Availability Statement

The original contributions presented in the study are included in the article/supplementary material, further inquiries can be directed to the corresponding author.

## Author Contributions

TY and FQ contributed to the conception and design and drafted the manuscript. TY, YW, and FQ extracted the data and evaluated the quality. YW, HL, and ZK verified the data. TY, FQ, YW, HL, and ZK contributed to the analysis and interpretation of the data. TY, FQ, YW, HL, and ZK revised it critically for important intellectual content. All authors have read and approved the final version of the manuscript.

## Funding

This research was funded by the Research Foundation for Advanced Talents of Beijing Sport University (3101033).

## Conflict of Interest

The authors declare that the research was conducted in the absence of any commercial or financial relationships that could be construed as a potential conflict of interest.

## Publisher's Note

All claims expressed in this article are solely those of the authors and do not necessarily represent those of their affiliated organizations, or those of the publisher, the editors and the reviewers. Any product that may be evaluated in this article, or claim that may be made by its manufacturer, is not guaranteed or endorsed by the publisher.
